# Trail pheromone identification in the ant *Crematogaster scutellaris*

**DOI:** 10.1038/s41598-024-58383-2

**Published:** 2024-04-03

**Authors:** Florencia Scarano, Daniele Giannetti, Francesco Trenti, Federico Giacomazzi, Jacopo Vigna, Graziano Guella, Donato A. Grasso, Albrecht Haase

**Affiliations:** 1 Center for Mind/Brain Sciences (CIMeC), University of Trento, Rovereto, Italy; 2 Department of Chemistry, Life Sciences and Environmental Sustainability (SCVSA), University of Parma, Parma, Italy; 3 Department of Physics, University of Trento, Trento, Italy

**Keywords:** Biochemistry, Physiology, Ecology

## Abstract

In this work, we identified the trail pheromone of the ant *Crematogaster scutellaris.* We combined gas chromatography–mass spectrometry analysis of extracts from the hind tibia, the location of the respective glands, with automated trail following assays. The study found tridecan-2-ol to be the strongest discriminator between hind tibia and other body part extracts. Tridecan-2-ol elicited trail-following behaviour at concentrations of 1 ng/µL. A separation of the enantiomers showed responses to (*R*)-tridecan-2-ol already at 0.001 ng/µL and only at a 1000-fold higher concentration for (*S*)-tridecan-2-ol, suggesting that only the *R* enantiomer is used by *C. scutellaris* in its natural environment. We also found strong behavioural responses to 2-dodecanol, a substance that was not detectable in the hind tibia extract of *C. scutellaris*, but which has been reported to be the trail pheromone of the related species *C. castanea.* We discuss the contribution of these results to the 'dissection and reconstruction' of strategies and mechanisms underlying the social organization of ants.

## Introduction

Ants are the dominant organisms in most terrestrial habitats and show impressive adaptive solutions to environmental modifications among social insects. Their ecological success is mainly supported by their complex social life and colony organization that allow ants to control their physical environment, efficiently exploit resources, and overcome competitors^1^. The complex organization of ant societies depends on the efficiency of different forms of communication, involving a great diversity of cues, such as chemical, acoustical, tactile, and visual. Olfaction is the principal sensory modality involved in integration and social coordination in ants. Hence, the complex interactions among nestmates in a colony are either partially or completely dependent upon semiochemicals^1,2^. Chemical communication mediated by pheromones is involved in many aspects of insect life with an extraordinary level of complexity reached in social insects, in particular ants. Specifically, of the almost 150 exocrine glands identified so far in social insects, 84 are present in ants and are mainly involved in communication contexts^3^. A substance produced by insects and used in intraspecific communication is categorized as either “primer” or “releaser” pheromones^3–5^. “Primer” pheromones are characterized by long-term physiological effects with no obvious immediate response. “Releaser” pheromones instead are categorized according to the behaviour they induce (*i.e.* sex pheromones, alarm pheromones, dispersal or spacing pheromones, trail pheromones, territorial and home range marking pheromones), evoking a rapid, non-permanent response^2,5,6^. In particular, chemical trail-laying communication represents the most prevalent form of recruitment behaviour in ants and allows group foragers to efficiently exploit conspicuous food sources as well as other important resources for the colony according to the habit and ecological needs of the species^1,7–11^. Trail-laying behaviour has been reported in a large number of ant genera. Yet, trail pheromones have been identified in a relatively small percentage of ant species and may consist of a variety of substances such as 2,3-dimethyl-5-(2-methylpropyl)-pyrazine, (*S*)-4-methyl-3-heptanone, 3,4-dihydro-8-hydroxy-3-methylisocoumarin^10,12^. Ant trail substances are produced by different glands mostly located in the distal part of the abdomen (the gaster), depending on the subfamily typically but not exclusively: Dufour's gland in Myrmicinae; hindgut in Formicinae and Ecitoninae; Pavan's gland in Dolichoderinae; sternal glands in Aneuretinae^10,12^. However, special locations for gland sources of trail pheromones have been reported, for example in different portions of the legs for a few species^13^. This is the case of the myrmicine genus *Crematogaster,* whose trail pheromone is released by the tibial glands^14,15^. The genus *Crematogaster* is characterized by worldwide distribution and comprises 780 species^16,17^. *Crematogaster scutellaris*^18^ is the most widespread arboreal ant in the Mediterranean area, up to Südtirol (Northern Italy). This species is considered one of the most highly-ranked competitors in Mediterranean ant communities. These ants usually form monogynous colonies with a lifespan of over 20 years with up to 20,000 workers. Moreover, they colonize and excavate different cavities on trees, dead wood, and galls^16,19,20^. Previous studies have shown that they can be a key study model in field and laboratory research and may have both positive and negative impacts in different anthropized habitats^21,22^. For these reasons, a better understanding of their communication system and in particular of their trail signals could have significant implications in both basic and applicative research. The only known trail-pheromone in the genus *Crematogaster* so far identified by both chemical analyses and trail-following behavioural bioassays is the alcohol (*R*)-2-dodecanol in the Afrotropical species *C. castanea*^23^.

The primary objective of our investigation was to analyse the secretion of the tibial glands of *C. scutellaris* responsible for the trail pheromone production and to then identify the compound/s eventually involved in trail-following by behavioural experiments. Given the presence of reservoirs in the front and hind legs^15^, we separately analysed components released from these glands. We tested the different components and their racemic forms in trail-following bioassays. We additionally assessed the effect of the trail pheromone of *C. castanea* (congeneric but fully allopatric with *C. scutellaris*) on *C. scutellaris* to evaluate the specificity of their trail-following behaviour^23^ and to look for potential interspecies effects.

## Results

### Chemical analysis of the tibial gland of *Crematogaster scutellaris*

**Figure 1:**
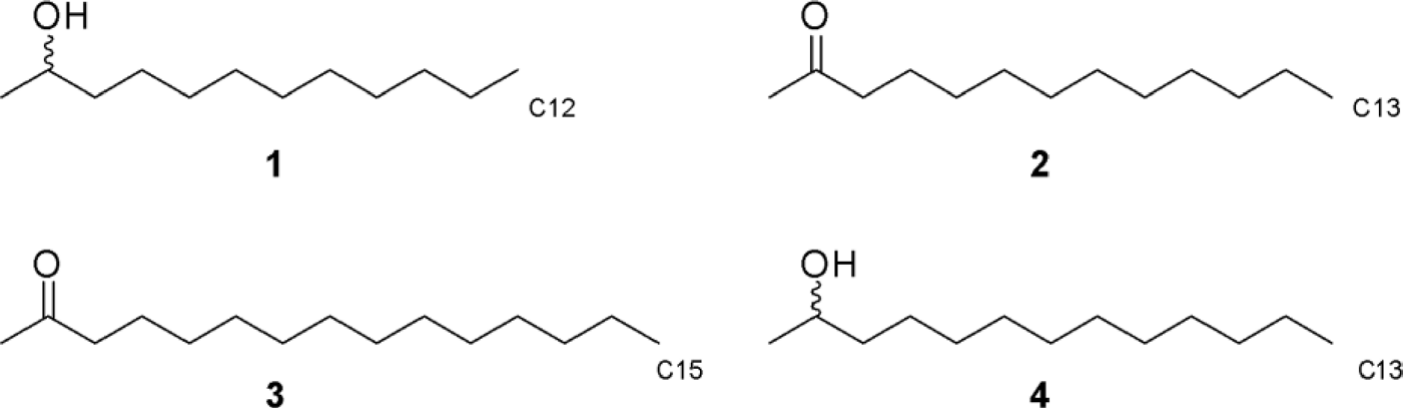
Standards selected for the trail pheromone investigation: 2-dodecanol (**1**); 2-tridecanone (**2**); 2-pentadecanone (**3**); and 2-tridecanol (**4**).

The GC–MS/FID data of the ants’ gland extracts were compared with the following standards: 2-dodecanol (**1**); 2-tridecanone (**2**); 2-pentadecanone (**3**); and 2-tridecanol (**4**) (Fig. 1). Notice that **1** and** 4** were obtained as racemic mixtures. 2-dodecanol was selected as a standard as it is reported to be the trail pheromone in the related ant species *C. castanea*^23^, and our behavioural experiments showed this compound to elicit a trail-following response in *C. scutellaris* as well. Besides that, we chose three similar chemicals differing by chain length and oxidation.

**Figure 2:**
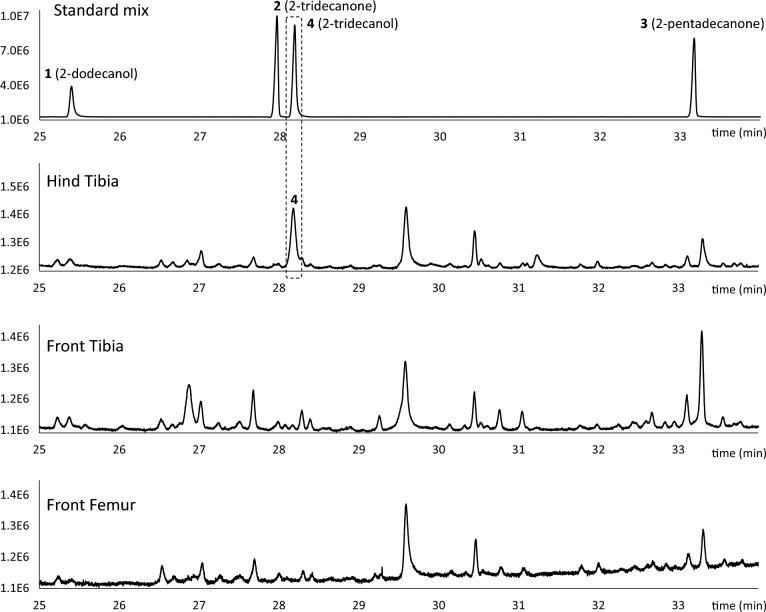
FID Chromatograms of standard mix (first top), and ant leg extracts (bottom three). The region between 25 and 35 min is highlighted to show the peak of interest (**4**) at RT 28.18 min.

**Figure 3:**
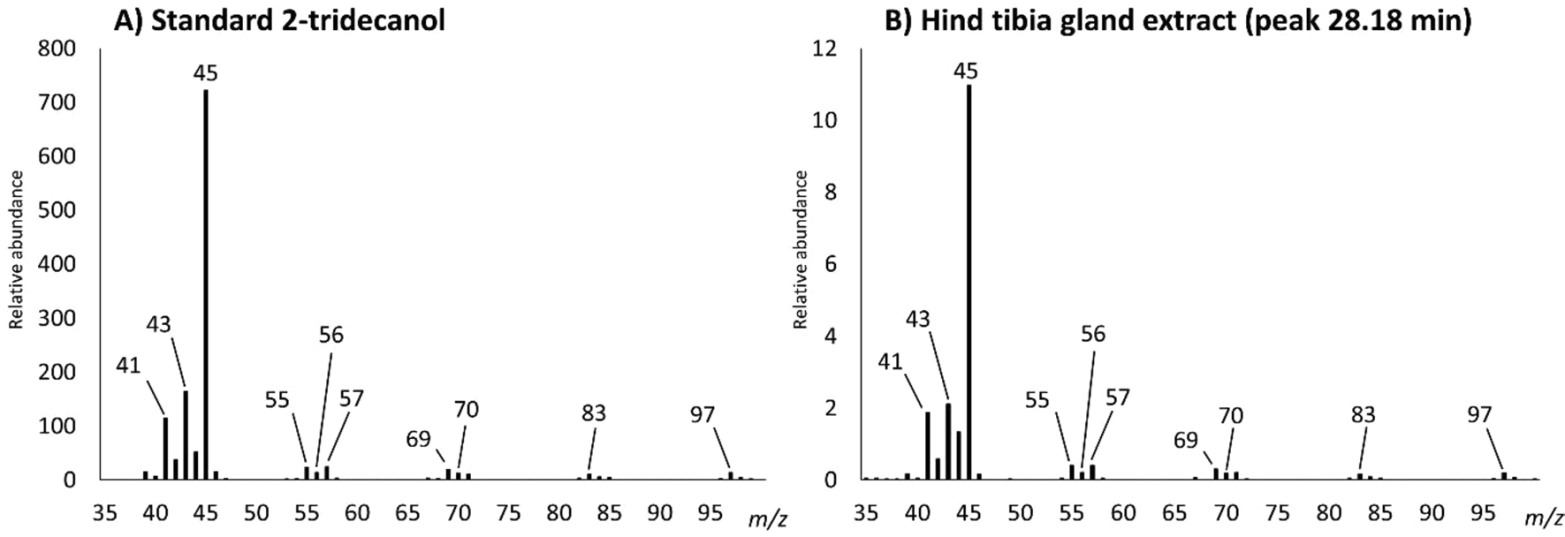
Matching MS spectra fingerprints of 2-tridecanol standard (**A**) and candidate peak at 28.18 min in the hind tibia extract (**B**).

Samples were analyzed by gas chromatography coupled to FID and MS detectors. The starting assumption was that the trail pheromone should be present in the hind tibia extract, as the gland responsible for its production is located in this limb^14,15^. Hence, the goal was to identify unique peaks in the hind tibia extract chromatogram, that were not present in the other limbs and could possibly align with the standards. Interestingly, the result was a single peak in the hind tibia extract at RT 28.18 min, which perfectly matched the peak of 2-tridecanol (**4**) both for retention time (Fig. 2) and MS fragmentation fingerprint (Fig. 3). No traces of 2-dodecanol (**1**) were found in any of the leg extracts. The minor peaks present in every leg extract between 25 and 25.5 min that apparently align with the 2-dodecanol standard (**1**), have in fact different retention times and completely unrelated ion fragmentation fingerprints compared to the 2-dodecanol standard, making them separate compounds. We are aware that Morgan reported 2-tridecanone (**2**) as the trail pheromone of *C. scutellaris* as an unpublished result^10^. However, our analyses of retention time and mass spectra of the trace peaks ruled out the presence of compounds (**2**) and (**3**) in any ant sample. No other peaks besides Hind tibia 28.18 min were therefore further investigated.

Not only the retention time between 2-tridecanol and the hind tibia peak at 28.18 min was the same, but also their ion fragmentation fingerprints matched perfectly (Fig. 3). This indicates that the trail pheromone of *C. scutellaris* is most likely 2-tridecanol.

### Comparison of behavioural responses to 2-dodecanol and 2-tridecanol

The trail-following responses of *C. scutellaris* ants were tested using 2-tridecanol, the putative trail pheromone, but also with 2-dodecanol, an active component of the trail pheromone of the congeneric species *C. castanea*^23^ not present in the trail secretion of *C. scutellaris*. This experiment allowed to evaluate the level of specificity of the trail signals (which are not always strict^1,24,25^) and an eventual conserved behavioural/perception mechanism still present in our focus species.

**Figure 4:**
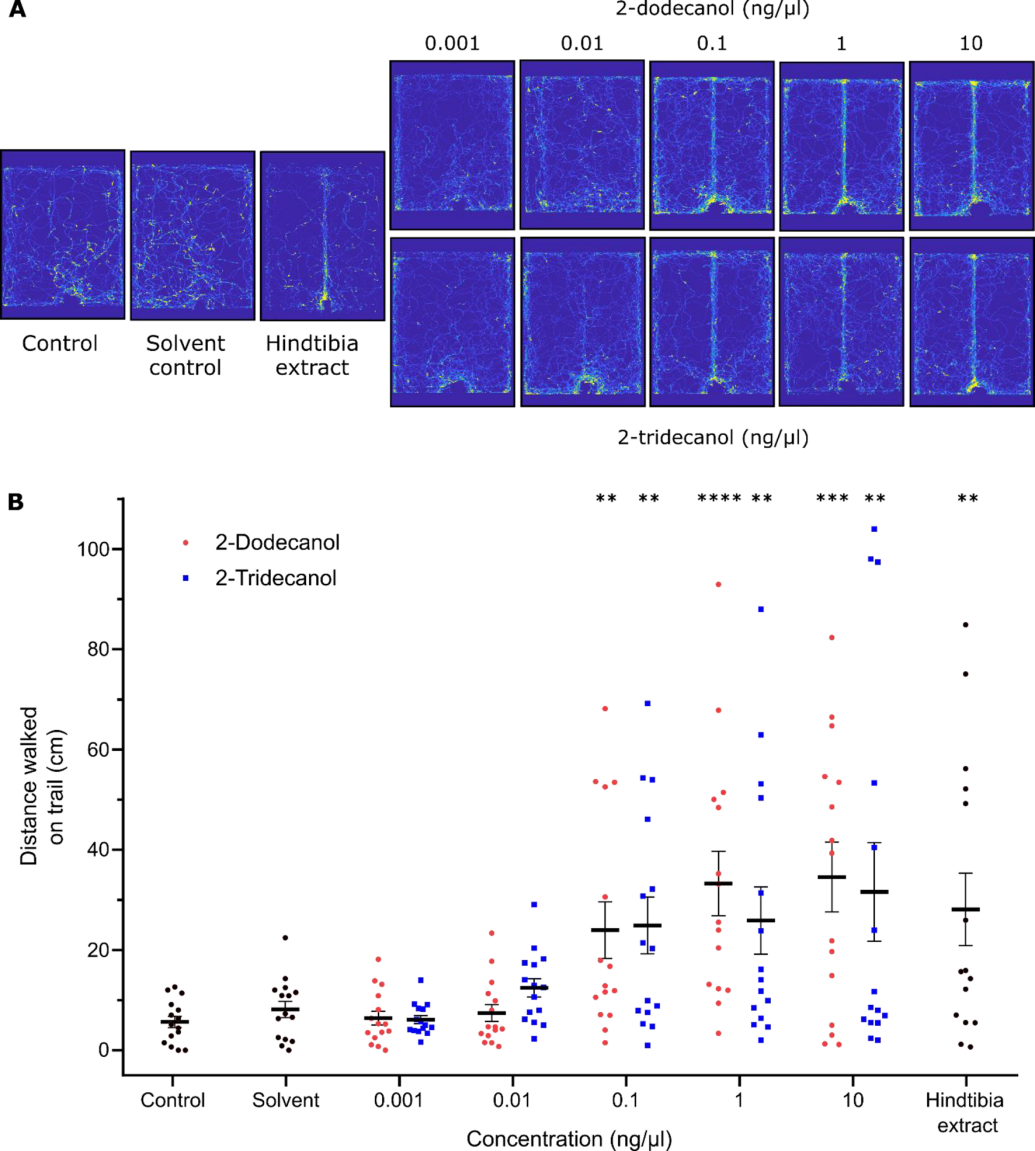
Distance walked by *C. scutellaris* ants on a trail. (**A**) Heatmaps for each experimental group (*n* = 15) showing the accumulated trajectories on the arena of all the tested ants. The yellow colour intensity represents the spatial passage probability. (**B**) The plots show Mean ± SEM distance as well as the results for single subjects as data points. Besides 2-dodecanol and 2-tridecanol**,** the solvent (hexane) was tested, as well as no odorant as control. Star labels represent the results of Dunnett’s multiple comparisons against the control: * *p* < 0.05, ** *p* < 0.01, *** *p* < 0.001, **** *p* < 0.001.

Figure 4 compares trail walking distances of *C. scutellaris* ants in response to 2-tridecanol, the novel compound that was found in its hind tibia extract, and 2-dodecanol, the trail pheromone of *C. castanea*. The heatmaps of the summed positions of all ants during the experiments clearly show that both compounds induce directional movement along the line to which they were applied (Fig. 4A). There is a clear contrast between the line and the rest of the arena, which excludes an unspecific excitatory effect of these compounds. Both chemical compounds evoked strong significant trail-walking responses at concentrations of 0.1 ng/µL or higher. Responses depended significantly on the preparation applied to the trail line (*F*(12,182) = 5.42; *p* < 0.0001). They were significantly stronger than the control for concentrations of 0.1 ng/µL or higher (Dunnett’s multiple comparisons, exact *p* values in Supplementary Table S1). There was no significant difference between the responses to the two active compounds at identical concentrations (unpaired *t*-test, *p* > 0.05 for each of the five concentrations, exact values in Supplementary Table S2). Responses to the hind tibia extract are shown as a positive control.

### Comparison of behavioural responses to 2-tridecanol enantiomers

Morgan et al*.* reported 2-dodecanol to be the trail pheromone in *C. castanea*^23^. In particular, one of its two enantiomers, (*R*)-(−)-dodecan-2-ol, resulted to be the main active component in the signalling. To test if chirality played a role also in the trial pheromone of *C. scutellaris,* the two enantiomers (*R*)-(−)-tridecan-2-ol **(R)-(−)4** and (*S*)-(+)-tridecan-2-ol **(S)-(+)4** where separated using L-(+)-mandelic acid as chiral derivatizing agent. Diastereoisomeric esters were synthesized by refluxing under acid catalysis a mixture of racemic alcohol and L-mandelic acid in toluene and then separated by column chromatography. Finally, hydrolysis of the separated esters with lithium hydroxide provided **(R)-(−)4** and **(S)-(+)4** enantiomers in good yields. The absolute configuration was assigned considering NMR chemical shift differences for mandelic esters^26^ (Supplementary Fig. S1) and by comparison with reported polarimetry data of hydrolyzed alcohols^27^.

**Figure 5:**
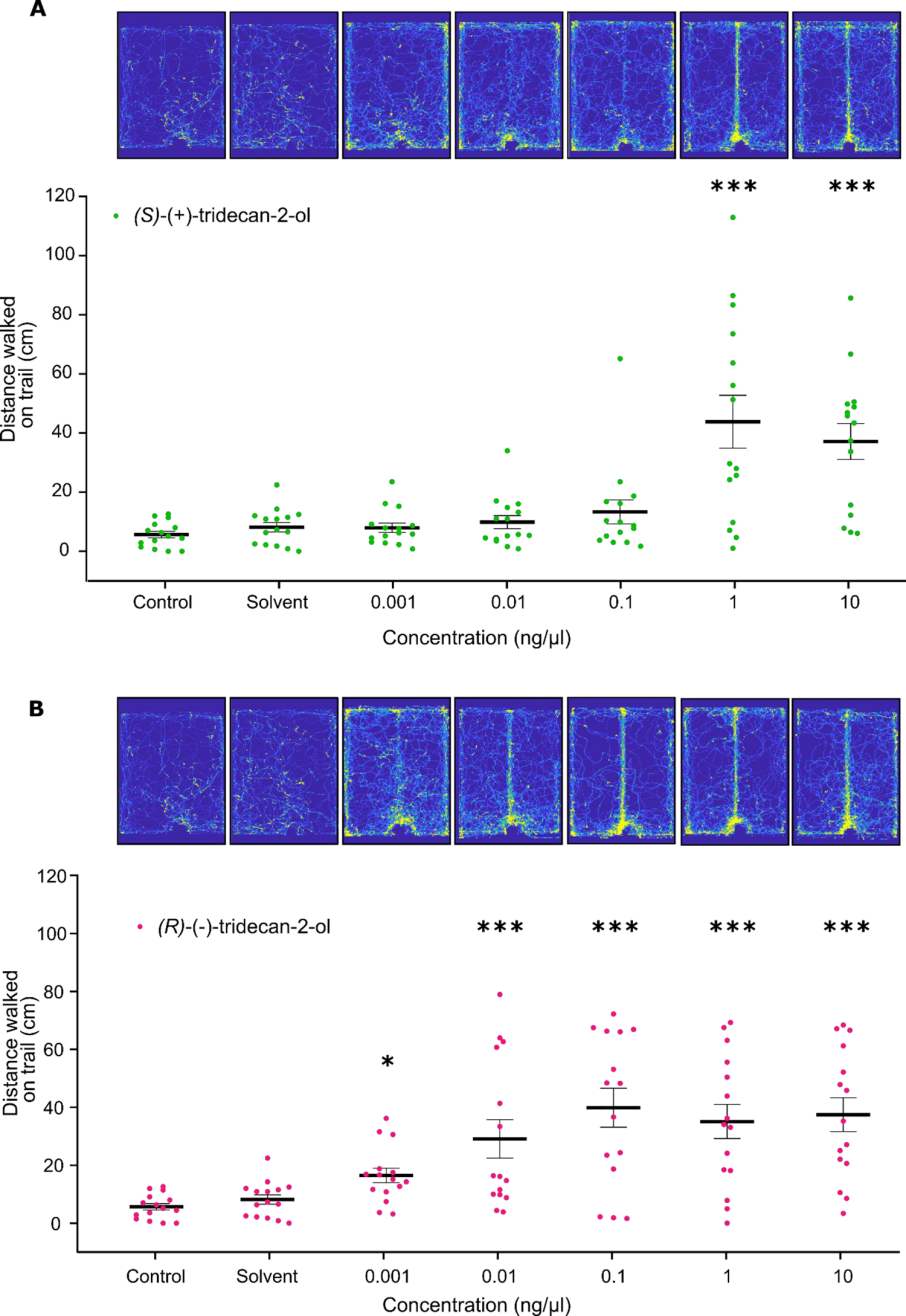
Distance walked by *C. scutellaris* ants on a trail of 2-tridecanol enantiomers (**A**) for (*S*)-(+)-tridecan-2-ol and (**B**) for (*R*)-(−)-tridecan-2-ol. Images on the top are heatmaps of each experimental group (*n* = 15) showing the accumulated trajectories of all tested ants. The yellow colour intensity represents the spatial passage probability. Below, Mean ± SEM distance walked on the trail are plotted. Ants exposed to (*R*)-(−)-tridecan-2-ol had a response significantly higher than the control at all tested concentrations. Ants exposed to (*S*)-(+)-tridecan-2-ol showed responses significantly higher than the control only for the two higher concentrations (1 and 10 ng/µL). Star labels represent the results of Dunnett’s multiple comparisons against the control: *p < 0.05, ***p < 0.001 (exact adjusted *p*-values in the Supplementary Table S1).

In the trail walking assay, both pure enantiomers were tested at 5 different concentrations (*n* = 15) (Fig. 5). Also here the behaviour depended on the preparation of the trail line (*F*(17,252) = 5.72, *p* < 0.0001). Ants responded significantly already to very low concentrations (0.001 ng/µL) of the *R* enantiomer, while responses to the *S* enantiomer were observed only at the two highest concentrations: 1 and 10 ng/µL (Dunnett’s multiple comparisons, adjusted *p*-values in Supplementary Table S3). This means that the *R* enantiomer evoked trail-following behaviour at concentrations that were three orders of magnitude lower than those at which the *S* enantiomer eventually did.

## Discussion

The basis of ant colony organization are very efficient communication systems. Among these, signals mediated by semiochemicals play a central role. The 'dissection and reconstruction' of the strategies and mechanisms underlying ant social organization, therefore, requires the study of chemical communication and, in particular, the identification of substances involved in it^8^. Chemical trail communication allows group foragers to exploit efficiently conspicuous food sources and represents the most prevalent form of recruitment behaviour in ants^1^. The presented research provides crucial information on the chemical trail communication systems of the acrobat ant *Crematogaster scutellaris*, a quite common arboreal ant widespread in the Mediterranean region^16^. By chemical analyses coupled with behavioural bioassays we identified for the first time the compound responsible for triggering trail-following response in these ants. Chemical trail signals seldom consist of a single substance produced by a single gland^1,8,12,28,29^. However, in the case of *C. scutellaris*, the composition of gland extract seems quite simple with a single chromatographic peak uniquely present in the extract of leg structures involved in trail-pheromone production and release (hind leg tibias) corresponding to the alcohol 2-tridecanol. Chemical analyses excluded the presence of the ketone 2-tridecanone in the gland extract and thus as a possible trail-pheromone of this species, contradicting unpublished results by Morgan^10^.

Behavioural tests using racemic 2-tridecanol confirmed this substance to be strongly active to induce, even at low concentrations, a typical trail-following behaviour by the ants.

For a more precise description of the chemical compound responsible for the trail-following response, we conducted a separation of the two enantiomers, (*S*)-(+)-tridecan-2-ol and (*R*)-(−)-tridecan-2-ol, by forming diastereomeric mandelic esters of 2-tridecanol. The two enantiomers were tested separately and (*R*)-(−)-tridecan-2-ol was found to activate ants already at a ca. 1000-fold lower concentration than (*S*)-(+)-tridecan-2-ol. The trail-following activity that was observed for (*S*)-(+)-tridecan-2-ol only at high dosages (1 ng/µL) could be attributed to residual trace amounts (4%) of (*R*)-(−)-tridecan-2-ol present in the sample.

Since (*R*)-(−)-tridecan-2-ol elicited a significantly stronger response compared to the *S* enantiomer, we argue that the former is the trail-following hormone that *C. scutellaris* ants produce in their hind tibial glands. However, we were not able to prove this experimentally, as too little of the hind tibia extract was available for the complex determination of the enantiomer composition by GC analysis using a chiral column. Hence the exact enantiomeric ratio of 2-tridecanol produced by *C. scutellaris* remains to be determined.

Interestingly, when testing 2-tridecanol as a racemic mixture, it elicited first significant effects only at concentrations of 0.1 ng/µL, which is 100-fold higher than when the (*R*)-(−)-tridecan-2-ol enantiomer was tested alone. A possible explanation for this discrepancy could be the physiological effect of mixture suppression, by which the neuronal activation produced in the insect olfactory network by a binary odour mixture (the two enantiomers) can be lower than the activation caused by each individual component^30^.

Complementary methods could help to solve the question of responsiveness to (*S*)-(−)-tridecan-2-ol in the future. The responses could be tested in the very periphery of the olfactory system via Electroantennography, but since this provides again an integrated response of all olfactory receptor neurons (ORNs), weak responses to high concentrations of the *S*-enantiomer could not be distinguished from strong responses to low concentrations of the *R*-enantiomer. If *S*- and *R*-compounds were to activate different ORNs, then calcium imaging in the ant antennal lobe could provide further insight^31,32^. But even then, several experiments with different mixing ratios might be necessary to avoid falsification by even the smallest impurities^33^.

Trail communication systems are strongly affected by a series of social and ecological influences that determine the properties of the signals. For example, different degrees of trail signal specificity could be influenced by intra- and interspecific competition, orientation and homing mechanisms, territorial recognition and advertisement^8,34^. The ants of the Myrmicinae subfamily studied so far show a strong variability of intra- and intergeneric trail specificity, ranging from a total or a partial specificity to a complete anonymity of signals^7,8,24^.

Workers of *C. scutellaris* follow the trails of *C. laestrygon*, but the latter always prefer their own trace^35^. *C. scutellaris* also displayed trail following responses to the trail pheromone of *C. auberti*. On the other hand, the trail pheromone of *C. scutellaris*, evoked responses in *C. auberti* and *C. laestrygon*, but not in *C. sordidula*^36^. In the present work, we showed that the trail-pheromone of *C. castanea* (2-dodecanol) induces a trail-following response in *C. scutellaris* workers even if the substance is not produced by these ants. This means a perceptual generalization seems to happen between 2-tridecanol and 2-dodecanol. On the other hand, no generalisation between the two enantiomers of 2-tridecanol could be observed. This can be explained by the fact that olfactory receptors in general are chiral themselves^37^, favouring greatly the binding of one enantiomer over the other, while the difference in one carbon in the chain length will less influence the binding strength.

The two species are fully allopatric, and this opens intriguing questions on both the proximate causes (*e.g.* mechanisms of perception, information processing, genetic bases etc*.*) and ultimate (evolutionary) processes underlying the phenomenon.

Species diverged in allopatry, from an evolutionary perspective, may have developed diverse recruitment and trail systems, leading to the use of different signals to convey the same messages. On the contrary, some overlap in signal production or perception among congeneric but allopatric species may have been maintained (or not selected against), since selection is not needed for strong signal divergence and specificity. This is especially valid if there are no other congeneric potential competitors around. Under these circumstances, it would be more important for a colony to maintain “privacy” on their trails with respect to other neighbouring colonies of the same species rather than species-specificity. Moreover, the colonial specificity of the signals also implies that a certain degree of privacy is maintained in relation to other species of ants present in the surroundings. In this context, it must be pointed out that trail specificity and 'ownership' could be provided in different ways and not only by depositing peculiar substances from a single gland, evoking specific trail-following behaviour^34^. Hence, it is important to distinguish between 'pure' artificial recruitment trails and natural foraging tracks, where trail signals may be used in conjunction with other pheromones and even other communication/perception channels (acoustical, visual cues)^7,24,28,38–41^.

*C. scutellaris* produce 2-tridecanol (likely only the *R* enantiomer as a pheromone triggering trail-following, but we hypothesize that as in other species of the same subfamily natural trails of *C. scutellaris* are not anonymously based only on this primer pheromone, but are probably added also with chemical markers from other glandular sources giving them colony specificity^42^.

The characterization of the trail-following pheromone of *C. scutellaris* not only fills a gap in the knowledge of the social biology and ecology of this common species but may also have significant applicative implications. Although occasionally, these ants may act as household pests^43^. In this context, having the possibility of interfering with their foraging ecology and communication strategies may result in reducing potential problems caused by their presence. On the other side, this species has been reported as extremely beneficial in certain environments, such as agroecosystems, where their presence, abundance, and activity as pest antagonists could be advantageous in biological control. In fact, these ants may act directly as plant defenders against pests^19,44–46^ or may have an indirect deterring effect on them due to their patrolling activity^45,47^. In this context, a trail-pheromone could be used as a direct control system allowing addressing the ants toward a specific target or (indirectly) deterring some pests from accessing suitable places for their attacks.

## Methods

### Colony collection and maintenance

Complete colonies (queen, brood, and workers) of *C. scutellaris* (*n* = 3) were collected in a field near the village of Fornoli (Northern Tuscany, Italy). All colonies were kept in artificial plastic nests (78 × 56 × 43 cm) with natural wood fragments and wet cotton balls to maintain humidity. Rearing conditions were: 25 ± 1 °C temperature, 50 ± 5% relative humidity, and a 16:8 h light:dark cycle. The colonies were fed with a sugar solution and larvae of *Galleria mellonella*.

### Ant legs extraction

Groups of ≈ 15 ants were placed in a 50 ml falcon tube which was then introduced in a freezer at – 20 °C for ≈ 5 min. Once the ants were dead, leg samples were dissected with clean tweezers under a binocular microscope keeping the leg segments of interest, which were immediately placed in an empty glass vial on dry ice. Via this procedure the following samples of three specimens were obtained: 400 hind tibias, 400 front tibias, and 400 front femurs. Samples were stored at – 80 °C and right before analysis, they were extracted by adding 50 µL of chloroform (CHCl_3_) to each, pipetting the heterogeneous solutions up and down with a syringe to mix them thoroughly. Due to the small volumes, the ant legs were not filtered off the solvent. They were stored at -20 °C to avoid evaporation of the solvent.

### Gas chromatography and standards preparation

The standards 2-dodecanol (**1**) (CAS: 10203-28-2); 2-tridecanone (**2**) (CAS: 593-08-8); 2-pentadecanone (**3**) (CAS: 2345-28-0); and 2-tridecanol (**4**) (CAS: 1653-31-2) were purchased at Sigma-Aldrich (Merck) and were prepared for gas chromatography at a final concentration of 10 ppm in chloroform. Standards and ant leg extracts were analysed by Trace GC Ultra GC (Finnigan) equipped with a flame ionization detector (FID) and coupled to a mass spectrometer Trace DSQ (Finnigan). A DB-5MS column (length 60 m, internal diameter 0.25 mm, film 0.25 µm; Agilent J&W) was used to separate the sample components and to annotate their retention time (RT). The following temperature program was set for the analysis: initial temperature 50 °C, reaching 275 °C with a rate of 5 °C/min (45 min ramp); the temperature was held at 275 °C for an additional 45 min to wash the column. The injector was heated at 300 °C and the carrier gas used was He (2.1 mL/min) in splitless mode. The MS transfer line was set at 250 °C; the MS detector was set to acquire in positive mode within a mass range of 35–300 m/z, starting acquisition at 8.5 min to avoid solvent ionization. Sample ionisation was achieved by electron impact (EI). The data were analyzed via the Thermo Xcalibur Qual Browser 2.2 SP1.48 using the NISTDEMO database. Long-chain alcohol standards were not recognized by the NISTDEMO database, while the 2-tridecanone standard was correctly identified. Therefore, the identification of compounds in the ant samples was based on retention time and ion fragmentation fingerprint.

### Enantiomer separation

All chemicals and reagents were purchased from Merck. Thin-layer chromatography (TLC) was performed using Merck silica gel F254, using KMnO_4_ solution as developing agent upon heating. Column chromatography was performed using Merck Si 45–60 µm as the stationary phase. The diastereoisomers and enantiomers were characterized by NMR, MS, FTIR, and polarimeter. NMR spectra were recorded on a Bruker-Avance 400 spectrometer using a 5-mm BBI probe ^1^H at 400 MHz and ^13^C at 100 MHz and calibrated using residual non-deuterated solvent for CDCl_3_ (relative to δ_H_ 7.27 and δ_C_ 77.0 ppm, respectively) with chemical shift values in ppm and *J* values in Hz. The following abbreviations were used to describe multiplicities: s = singlet, d = doublet, t = triplet, m = multiplet, br = broad. NMR data were analyzed using the BrukerTopspin software version 3.6.1. Electrospray ionisation ESI(+)MS mass spectra were recorded using a Bruker Esquire spectrometer by direct infusion of a methanol solution (source temperature 300 °C, drying gas N_2_, 4 L/min, scan range *m*/*z* 100:1000). Infrared spectra were recorded using a Jasco FT/IR-4700 spectrometer. Polarimetric data were obtained with a Jasco DIP-181 apparatus, at 25 °C using Na source. All compounds were obtained > 99% pure by NMR analysis. Yields are given on recovered purified fractions.

### Esterification reaction for the synthesis of mandelic acid esters

Compound **4** (100 mg, 0.5 mmol, 1 eq.) was added to a stirred solution of L-(+)-mandelic acid (152 mg, 1 mmol, 2 eq.) and *p*-TsOH·H_2_O (10 mg, 0.1 eq.) in toluene (2 mL) and the temperature was raised to 110 °C for 3 h. The solution was washed with 10 mL Na_2_CO_3_ 2 M and the aqueous phase was extracted with dichloromethane (2×). The organic phases were washed with brine, dried over anhydrous Na_2_SO_4_ and evaporated under reduced pressure. The NMR analysis shows the formation of *RS* and *SS* diastereomers in 1:1 ratio (80% conversion). The reaction products were purified by column chromatography on silica gel using dichloromethane/hexane 9:1 as eluent mixture.

(*R*)-tridecan-2-yl (*S*)-2-hydroxy-2-phenylacetate. Colourless oil; 63 mg (40%); *R*_*f*_ = 0.62 (CH_2_Cl_2_/hexane = 9:1); [*α*]_D_ =  + 38° ± 1° (*c* 1.0, CH_2_Cl_2_); ^1^H-NMR (400 MHz, CDCl_3_) *δ*: 7.46–7.40 (m, 2H), 7.39–7.30 (m, 3H), 5.15 (s, 1H), 5.04–4.94 (m, 1H), 3.56 (br s, 1H), 1.69–1.57 (m, 1H), 1.55–1.45 (m, 1H), 1.29 (s, 18H), 1.06 (d, *J* = 6.5 Hz, 3H), 0.92 (t, *J* = 7.0 Hz, 3H); ^13^C-NMR (100 MHz, CDCl_3_) δ: 173.3, 138.5, 128.4 (2C), 128.2, 126.4 (2C), 73.5, 72.8, 35.6, 31.9, 29.6, 29.6, 29.5, 29.5, 29.3, 29.3, 25.2, 22.6, 19.4, 14.1; FT-IR: br 3499, 2921, 2853, 1729, 1493, 1453, 1380, 1261, 1171, 1125, 1093, 1065, 1065, 1027, 729, 694 cm^-1^; ESI(+)MS: *m/z* 357 [M + Na]^+^; d.e. > 96%.

(*S*)-tridecan-2-yl (*S*)-2-hydroxy-2-phenylacetate. White powder; 51 mg (30%); *R*_*f*_ = 0.56 (CH_2_Cl_2_/hexane = 9:1); [*α*]_D_ =  + 94° ± 1° (*c* 1.0, CH_2_Cl_2_); ^1^H-NMR (400 MHz, CDCl_3_) *δ*: 7.46–7.40 (m, 2H), 7.38–7.29 (m, 3H), 5.13 (s, 1H), 5.00–4.91 (m, 1H), 3.62 (br s, 1H), 1.48–1.36 (m, 2H), 1.34–1.18 (m, 13H), 1.18–1.11 (s, 2H), 1.10–1.00 (m, 4H), 0.96–0.90 (m, 2H), 0.90 (t, *J* = 6.6 Hz, 3H); ^13^C-NMR (100 MHz, CDCl_3_) δ: 173.5, 138.7, 128.4 (2C), 128.3, 126.5 (2C), 73.4, 72.9, 35.7, 31.9, 29.6, 29.6, 29.4, 29.3 (2C), 29.1, 24.7, 22.7, 19.9, 14.1; FT-IR: br 3436, 2918, 2850, 2395, 1822, 1467, 1453, 1380, 1261, 1212, 1184, 1125, 1095, 1065, 1029, 729, 692, 608, 586, 502 cm^-1^; ESI(+)MS: *m*/*z* 357 [M + Na]^+^; d.e. > 99%.

### General procedure for the hydrolysis of esters

*RS* or *SS* diastereoisomer (30 mg, 0.09 mmol, 1 eq.) was dissolved in 1 mL of tetrahydrofuran and LiOH∙H_2_O (15 mg, 4 eq.) dissolved in 0.3 mL of H_2_O/CH_3_OH 1:2 was added. The solution was stirred at room temperature for 2 h and then concentrated under reduced pressure. The residue was dissolved in H_2_O and extracted with dichloromethane (3×). The organic phases were washed with brine, dried over anhydrous Na_2_SO_4_ and evaporated under reduced pressure. The crudes were purified by column chromatography on silica gel using dichloromethane as eluent. The enantiomers were obtained as colourless oil; *R*_*f*_ = 0.41 (CH_2_Cl_2_ 100%); ^1^H-NMR (400 MHz, CDCl_3_) δ: 3.85–3.75 (m, 1H), 1.50–1.38 (m, 4H), 1.33–1.25 (m, 17H), 1.19 (d, *J* = 6.0 Hz, 3H) 0.89 (t, *J* = 6.6 Hz, 3H); ^13^C-NMR (100 MHz, CDCl_3_) *δ*: 68.0, 39.2, 31.8, 29.5 (5C), 29.2, 25.6, 23.3, 22.5, 13.9; FT-IR: br 3332, 2958, 2921, 2850, 1462, 1373, 1137, 1113, 940, 720 cm^−1^.

**(S)(+)4**: (*S*)-(+)-tridecan-2-ol. 15 mg (85%); [*α*]_D_ =  + 7.1° ± 0.9° (*c* 1.0, C_2_H_5_OH); e.e. 96%.

**(R)(−)4**: (*R*)-(−)-tridecan-2-ol. 17 mg (97%); [*α*]_D_ =  − 7.5° ± 0.5° (*c* 1.0, C_2_H_5_OH); e.e. > 99%.

### Behavioural experiments

The arena consisted of an open plastic box (20 × 14 × 5 cm) with a plastic tube (inner diameter: 6 mm; outer diameter: 8 mm) embedded in one of the shorter side walls. For each experiment, a new sheet of paper (16 × 10 cm) was placed inside the box. The paper was traversed lengthwise by a toner grey line (hereafter trail-line) onto which the different test solutions were applied (Supplementary Videos S1 and S2).

Solutions of 2-dodecanol, 2-tridecanol, (*R*)-(−)-tridecan-2-ol and (*S*)-(+)-tridecan-2-ol were prepared in hexane at concentrations of 10^–3^, 10^–2^, 10^–1^, 10^0^, 10^1^ ng/µL, respectively. Hind tibiae extract was obtained by adding 100 hind tibiae to a vial containing 100 µL of hexane and allowing it to infuse overnight in the refrigerator. Two controls were performed: one with only hexane solvent and one without any additive.

Before each experiment, the end of the tube in the box was sealed with a small piece of cotton and an ant was carefully taken by the thorax with soft tweezers and placed in the tube. Then, the outer end of the tube was also sealed with a small piece of cotton. To recover from the stress caused by these manipulations, each ant remained in the tube for 2 min previous to the experiment. During this time, 10 µL of one of the test solutions was distributed along the trail line using a micropipette with a long thin plastic tip (capillary microloader from Eppendorf). The solvent was allowed to evaporate, and the paper was placed in the plastic box. The cotton sealing the inner end of the tube was then removed to allow the ant to enter the experimental arena. If an ant was taking longer than 2 min to leave the tube, the experiment was cancelled and the ant was considered non-responsive. Once the ant left the tube, the experiment started and the ant’s activity was video-recorded for 3 min (Supplementary Videos S1 and S2). The camera (HD high-speed camcorder JVC GC-PX100) was placed vertically on a tripod at 61 cm above the arena. The videos were recorded at a resolution of 720 × 1280 pixels and 25 frames/s. After every experiment, the paper was discarded and all surfaces of the box and the tube were cleaned with a wet paper towel and allowed to dry before the new experiment started. Each solution was tested in *n* = 15 ants individually, 5 from each of the three colonies. Each ant was tested only once with a single substance at a single concentration.

### Data analysis

The videos were analysed using a specially written code in MATLAB (https://github.com/NeurophysicsTrento/Ant-Trail-Following). To shorten the analysis time, all the videos were compressed by binning 4 × 4 pixels and 4 frames, resulting in a spatial resolution of about 1 mm and a temporal resolution of 160 ms. After the experimenter has graphically marked the trail line via the user interface, the ant is identified by automatic image segmentation and its relative distance to the trail line is calculated. The ant was considered walking on the trail if the distance between its body centre and the trail line was less than 5 mm. On this basis, the distance each ant walked on the trail during the 3 min experiment was calculated. This then serves as a measure for the directional movement induced by the substance applied to the trail line^10^ (Supplementary Videos S1 and S2).

For the statistical analysis, the walking distances were transformed to $Y={\text{ln}}(y+1)$ to fulfil parametric assumptions. A one-way ANOVA was used to test a general dependence of the line walking distance on the substance applied to the line. Dunnett’s multiple comparisons were utilized to compare individual concentrations of a compound against the control. Unpaired *t*-tests were used for comparisons between compounds at identical concentrations.

### Supplementary Information

Supplementary Video S1.

Supplementary Video S2.

Supplementary Information.

## Data Availability

The analysis code is publically available from https://github.com/NeurophysicsTrento/Ant-Trail-Following. The datasets will be provided by the corresponding author upon reasonable request.
