# Supplementary material for: Trail pheromone identification in the ant Crematogaster scutellaris
Source: Sci Rep. 2024 Apr 3;14:7883. doi: 10.1038/s41598-024-58383-2 (PMC10991323; doi:10.1038/s41598-024-58383-2)
Supplement: Supplementary file 3 — Supplementary Information. [file 41598_2024_58383_MOESM3_ESM.pdf]

Supplementary information

Supplementary Table S1. Adjusted *p*-values for Dunnett's multiple comparisons test: Different Substances/concentrations vs. control. Significant results are labelled red.

Dunnett's multiple comparisons test	Mean Diff.	95.00% CI of diff.	Adjusted *p*-value
Control vs. Solvent	-0.31	-1.3 to 0.67	0.9779
Control vs. Dodec 0.001	-0.11	-1.1 to 0.86	0.9996
Control vs. Dodec 0.01	-0.25	-1.3 to 0.72	0.9921
Control vs. Dodec 0.1	-1.2	-2.2 to -0.24	0.0059
Control vs. Dodec 1	-1.7	-2.7 to -0.68	<0.0001
Control vs. Dodec 10	-1.5	-2.5 to -0.48	0.0005
Control vs. Tridec 0.001	-0.27	-1.3 to 0.71	0.9907
Control vs. Tridec 0.01	-0.85	-1.9 to 0.13	0.1199
Control vs. Tridec 0.1	-1.2	-2.2 to -0.25	0.0053
Control vs. Tridec 1	-1.2	-2.3 to -0.26	0.0048
Control vs. Tridec 10	-1.2	-2.2 to -0.21	0.0080
Control vs. Hindtibia extract	-1.2	-2.2 to -0.22	0.0070

Supplementary Table S2. Multiple *t*-tests: 2-dodecanol vs. 2-tridecanol at specific concentrations.

	p-value	Mean of 2-dodecanol	Mean of 2-tridecanol	Difference	SE of difference	*df*
Control	>0.99	5.7	5.7	0	1.6	28
Solvent	>0.99	8.1	8.1	0	2.3	28
0.001	0.85	6.4	6.1	0.32	1.7	28
0.01	0.055	7.4	12.5	-5.0	2.6	28
0.1	0.91	23.0	24.9	-0.93	8.0	28
1	0.44	33.3	25.9	7.4	9.3	28
10	0.81	35	32	3.0	12	28

Supplementary Table S3. Adjusted *p*-values for Dunnett's multiple comparisons test: Different Enantiomers/concentrations vs. control. Significant results are labelled red.

Dunnett's multiple comparisons test	Mean Diff.	95.00% CI of diff.	Adjusted *p*-value
Control vs. Solvent	-0.31	-1.3 to 0.68	0.99
Control vs. R 0.001	-1.1	-2.0 to -0.10	0.021
Control vs. R 0.01	-1.4	-2.5 to -0.43	0.0007
Control vs. R 0.1	-1.7	-2.7 to -0.68	<0.0001
Control vs. R 1	-1.6	-2.6 to -0.62	<0.0001
Control vs. R 10	-1.8	-2.8 to -0.81	<0.0001
Control vs. S 0.001	-0.38	-1.4 to 0.61	0.96
Control vs. S 0.01	-0.50	-1.5 to 0.49	0.76
Control vs. S 0.1	-0.70	-1.7 to 0.30	0.35
Control vs. S 1	-1.7	-2.8 to -0.76	<0.0001
Control vs. S 10	-1.8	-2.8 to -0.78	<0.0001
Control vs. Hindtibia extract	-1.2	-2.2 to -0.21	0.0075

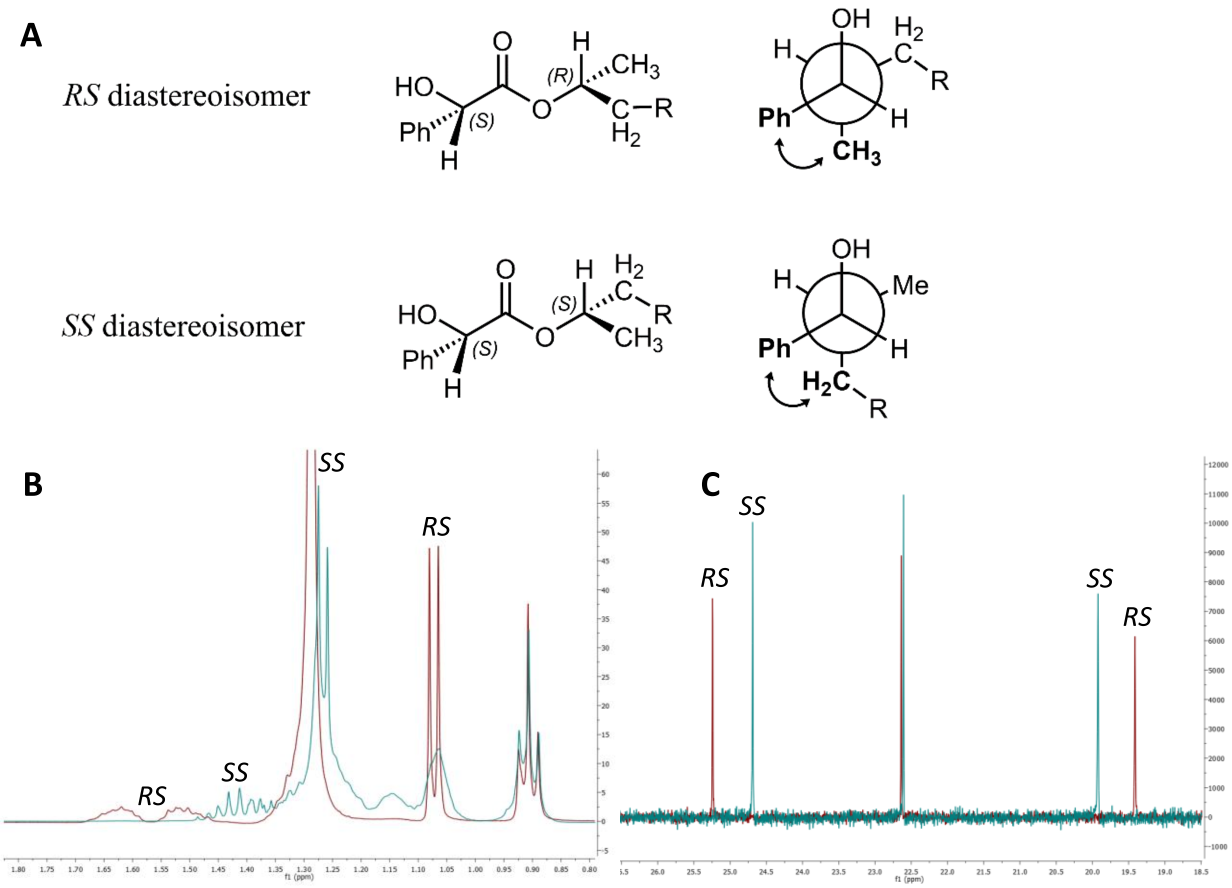

Supplementary Figure S1. Determination by NMR analysis[1] of absolute stereochemistry for the esters formed by the reaction of racemic 2-tridecanol and (+)-(*S*)-mandelic acid. (**A)** Anisotropic shielding is highlighted in Newman projections for *RS* and *SS* diastereoisomes. The proximity of the phenyl ring causes a shielding effect to the CH_3 group in *RS* diastereoisomer and to the CH_2-R group in the *SS* diastereoisomer. **(B)** ^{1}H-NMR spectra of *RS* (red) and *SS* (blue) esters. CH_3 signal of *RS* (d, 1.06 ppm) and CH_2-R signal of *SS* (m, 1.48-1.36 ppm) are shielded due to the proximity of the phenyl group. On the contrary, CH_3 signal of *SS* (d, 1.26 ppm) and CH_2-R signals of *RS* (m, 1.69-1.57 ppm and m, 1.55-1.45 ppm) result more deshielded. **(C)** ^{13}C-NMR spectra of *RS* (red) and *SS* (blue) esters. CH_2-R signal of *SS* (24.7 ppm) and CH_3 signal of *RS* ester (19.4 ppm) are more shielded in comparison with their signals in the other diastereoisomer.

1. Salvatore, B. A. Physical methods and techniques. NMR Spectroscopy. *Annu. Reports Sect. 'B' (Organic Chem.* **95**, 395–417 (1999).

Supplementary Video S1: Exemplary video of an ant exposed to a trail of 2-tridecanol at a concentration of 1 ng/µl. Displayed parameters are time in frames (≈160 ms frame time), the *x*,*y*-position in pixels (≈1 mm pixel size), and the perpendicular distance to the trail line in cm.

Supplementary Video S2: Control experiment without any substance applied to the trail. Displayed parameters are time in frames (≈160 ms frame time), the *x*,*y*-position in pixels (≈1 mm pixel size), and the perpendicular distance to the trail line in cm.
